# Changes in Retinal Nerve Fiber and Ganglion Cell Layers After Chemical Injury: A Prospective Study

**DOI:** 10.3390/jcm14155601

**Published:** 2025-08-07

**Authors:** Justina Skruodyte, Justina Olechnovic, Pranas Serpytis

**Affiliations:** 1 Faculty of Medicine, Vilnius University, LT-01131 Vilnius, Lithuania; 2 Eye Diseases Department, Republican Vilnius University Hospital, LT-04130 Vilnius, Lithuania; 3 Institute of Clinical Medicine, Faculty of Medicine, Vilnius University, LT-01131 Vilnius, Lithuania

**Keywords:** chemical eye burns, chemical injury, retinal ganglial cells, retinal nerve fiber layer, retinal structural changes

## Abstract

**Background:** Chemical eye burns are a serious ophthalmic emergency that can lead to permanent vision loss in severe cases. This study aims to evaluate structural changes in the posterior segment of the eye in individuals who have experienced chemical burns. **Methods:** The study included 64 eyes from 54 patients with chemical burns (chemical burn group) and 87 healthy eyes from 87 subjects (control group), matched by age and sex. Patients had confirmed burns with limbal ischemia, no glaucoma, normal intraocular pressure, and no major ocular or systemic diseases. Burned eyes were examined during the acute phase and again at 3 months, with some followed up at 6 months if significant retinal asymmetry was detected. Retinal nerve fiber layer (RNFL) thickness was assessed in four quadrants, and ganglion cell complex (GCL++) thickness was analyzed using automated segmentation of optical coherence tomography (OCT) maps. **Results:** This study compared measurements between the burn group, the control group, and timepoints. OCT analysis revealed no significant difference in total RNFL thickness between burn patients and controls (mean difference: −1.14 µm, 95% CI: −3.92 to 1.64). Similarly, GCL++ thickness did not differ significantly between groups (mean difference: −0.97 µm, 95% CI: −3.31 to 1.37). At 6-month follow-up, a non-significant decline in both RNFL and GCL++ thicknesses was observed. Logistic regression identified higher Dua grade as an independent predictor of RNFL thinning (OR: 4.816, 95% CI: 1.103–21.030; *p* = 0.037). Patients with severe ocular chemical burns (Dua grade ≥ 3) demonstrated reduced RNFL thickness in all quadrants compared to healthy controls. The most pronounced reductions were observed in the nasal and superior quadrants (*p* = 0.007 and *p* = 0.069, respectively); however, after applying Bonferroni correction for multiple comparisons, only the difference in the nasal quadrant remained statistically significant (*adjusted p* = 0.035). **Conclusions:** Although overall RNFL and GCL++ thicknesses did not differ significantly between burn patients and healthy controls, patients with severe ocular chemical burns (Dua grade ≥ 3) showed a significant reduction in RNFL thickness, in the nasal quadrant. Higher Dua grade was identified as an independent predictor of RNFL thinning. These findings suggest a potential association between burn severity and posterior segment changes, highlighting the need for further longitudinal studies with larger cohorts.

## 1. Introduction

Chemical eye burns are a serious ophthalmic emergency that can lead to permanent vision loss in severe cases. The severity of a chemical eye burns depends on several factors: the type and strength of the chemical, duration of exposure, and promptness of initial treatment. These injuries are typically caused by alkaline and acidic substances. However, alkali agents damage the eye more due to their ability to penetrate ocular tissues rapidly. Compared to acid burns, alkali burns occur more frequently. Their widespread use in homes and businesses is the primary contributing factor [1]. Immediate irrigation of the eye and appropriate medical intervention help reduce the harm and improve prognosis [2].

While most cases are mild and not sight-threatening, there are occurrences where the structures of the anterior segment are significantly damaged, and long-term complications such as glaucoma can occur. Chronic and progressive glaucomatous changes in the optic nerve and ganglion cells (GCs) can lead to irreversible loss of visual function. A series of reports describing de novo glaucoma as a complication after anterior segment injuries without documented elevation of intraocular pressure (IOP) after the injury encouraged scientists to search for alternative pathogenesis pathways of the process [3]. Experiments were conducted on rabbits and mice, inducing alkali burns on the corneas to determine which substances were involved and the precise location of the damage. It was shown that proinflammatory cytokines such as tumor necrosis factor (TNF)-α, TNF-R1, TNF-R2, interleukin (IL)-1β, and matrix metalloproteinase-9 (MMP-9) are produced by the iris and ciliary body and diffuse into the posterior segment [4]. This triggers monocytes from the periphery to enter the retina. Inside the retina, they change into macrophages and acquire a morphology that resembles microglia. These cells move into the ganglion cell layer (GCL), inner plexiform layer (IPL), inner nuclear layer (INL), and outer plexiform layer (OPL). Blood-derived monocytes stay in the retina permanently after injury. Unlike the yolk-sac-derived microglial cells they mimic, peripheral CX3CR1⁺ cells remain proinflammatory, actively phagocytose neuroretinal tissue for months after engraftment, and become resistant to mediators that typically induce microglial depletion [5]. These abnormal cell features lead to chronic and progressive damage to the GC and optic nerve axons. Experiments on animals aimed at modulating inflammation and its consequences for the posterior eye pole were performed. Most of these studies reported that corticosteroid prophylaxis had no effect in reducing GC and optic nerve axon loss, most likely due to their inhibition being attributed to other molecules released by injured tissues, such as interferon-γ [6]. However, immunomodulators used in proper doses show promising results, as they significantly reduce the loss of GC and optic nerve axons [6,7]. As described in the literature, a cascade of pathological and irreversible events leading to GC and optic nerve axon damage begins immediately after a chemical injury to the cornea. In mice, cytokines such as TNF-α are highly upregulated within 24 hours after a corneal alkali burn [6,8]. Prompt anti-TNF-α therapy has been shown to suppress inflammation and protect the retina from apoptotic changes [4]. This means that early prevention through the administration of appropriate medications in the emergency department could be highly effective. Moreover, similar GC and optic nerve axon damage has been documented not only after severe chemical corneal injuries and intense inflammation in the anterior segment of the eye but also following other interventions such as keratoplasty, ocular trauma models, or even a single full-thickness corneal suture [6].

While changes in the posterior pole have only been documented in animal experiments, further investigations are needed to determine whether such changes also occur in human eyes following chemical injuries in vivo. This study examined the demographic and clinical characteristics of patients with chemical eye burns, assessed OCT measurements, and aimed to improve recognition of posterior segment involvement in chemical ocular injuries.

## 2. Methods

This prospective study was performed at the Republican Vilnius University Hospital in Lithuania between July 2023 and April 2025. The study was conducted in accordance with the Declaration of Helsinki, and the protocol was approved by the Vilnius Regional Biomedical Research Ethics Committee (No. 2023/5-1520-976). Informed consent was obtained from all subjects. The study included 64 eyes from 54 patients with chemical burns (chemical burn group) and 87 healthy eyes from 87 subjects (control group). A power analysis was conducted using G*Power software (version 3.1) to assess whether the study was sufficiently powered to detect meaningful differences between groups. Given the total sample size of 151 eyes (64 in the chemical burn group and 87 in the control group), the study had 80% power to detect a medium effect size (d = 0.5) at a significance level of α = 0.05. Inclusion criteria for patients with eye burns were as follows: (1) eye burns diagnosed by an ophthalmologist; (2) patients who agreed to return for follow-up 3–6 months after a chemical burn at the Republican Vilnius University Hospital; (3) no history of glaucoma; (4) normal IOP ≤ 21 mmHg; (5) limbal ischemia observed at the time of the ocular burn; (6) no ocular diseases present, except for mild refractive errors (hyperopia up to +3.00 D, myopia up to −3.00 D, and astigmatism ≤ 1.50 D); and (7) no history of systemic or infectious disorders (e.g., poorly controlled diabetes, Alzheimer’s disease). The burn group was examined during the acute phase and again three months after the injury. If significantly asymmetry between the eyes was observed, the subjects were invited for an additional follow-up examination three months later (i.e., six months after the injury) to assess any progression. According to the literature, differences in RNFL thickness greater than 9 μm and GCL thickness differences greater than 4.5 μm should be considered significantly asymmetrical [9,10,11,12]. To address the potential violation of the independence assumption due to the inclusion of both eyes from some participants, a sensitivity analysis was performed using one randomly selected eye per participant. The statistical results remained consistent with the original analysis, with no significant changes observed. Control subjects were randomly selected from hospital visitors using age- and sex-matching criteria. Inclusion in the control group required healthy eyes with no ophthalmological or systemic diseases, except for mild refractive errors (hyperopia up to +3.00 D, myopia up to −3.00 D, and astigmatism ≤ 1.50 D); as reported in previous studies [13,14]. Prior to inclusion, all individuals underwent a standardized medical interview, during which they were specifically asked about systemic conditions such as diabetes mellitus, Alzheimer’s disease, arterial hypertension, cardiovascular disease, autoimmune disorders, and chronic medication use. Only individuals who reported no known systemic illnesses and showed no clinical signs of ocular pathology were included. The right eye was generally selected for analysis; however, if the right eye was ineligible (e.g., post trauma), the left eye was used instead. Control subjects underwent the same ophthalmological examinations as patients with chemical eye injuries. All participants were older than 18 years. Ocular burn severity was classified according to the Dua classification system [15,16]. In this system, all grades were analyzed except Grade I since only subjects with limbal ischemia were included, and Grade VI, which was not represented in the study population. The posterior segment was imaged using OCT (DRI-OCT Triton, Topcon, Tokyo, Japan). The early treatment diabetic retinopathy study (EDTRS) grid (6 × 6 mm) was used to divide the RNFL into the thickness maps (µm) to gain the values for each sector. The grid was composed of four quadrants (superior, inferior, nasal, temporal). GCL++ thickness—measured from the inner limiting membrane to the inner plexiform layer/inner nuclear layer interface—was analyzed using the automated segmentation OCT map. Only scans with a quality score above 50 (for OCT structural measurements), based on a scale ranging from 0 (“poor quality”) to 100 (“excellent quality”), were included, in accordance with previous studies [17,18]. Images with motion artifacts, poor centration, or segmentation errors were excluded. All scans were performed by the same experienced operator. OCT was performed at 3 and, if needed, 6 months. The collected data were processed and analyzed using IBM SPSS Statistics version 27.0. Qualitative variables were presented as frequencies and percentages. The mean ± standard deviation and range of RNFL and GCL were calculated at 3 and 6 months. The normality of the data distribution was assessed using the Shapiro–Wilk and Kolmogorov–Smirnov tests. Student *t*-tests for independent and paired samples were used when normality assumptions were met. Otherwise, the Mann–Whitney U test was used for two independent samples and the Wilcoxon paired-rank test was used for two paired samples. For normally distributed quantitative variables, Pearson’s correlation coefficient was used to evaluate relationships. For non-normally distributed data, Spearman’s rank correlation was applied. Binary logistic regression was used to predict RNFL and GCL thinning. Results with a *p*-value less than 0.05 (*p* < 0.05) were considered to be statistically significant. To control for the increased risk of Type I error due to multiple comparisons of RNFL thickness across the four retinal quadrants, a Bonferroni correction was applied. Bonferroni-adjusted *p*-values were reported where applicable.

## 3. Results

**Figure 1:**
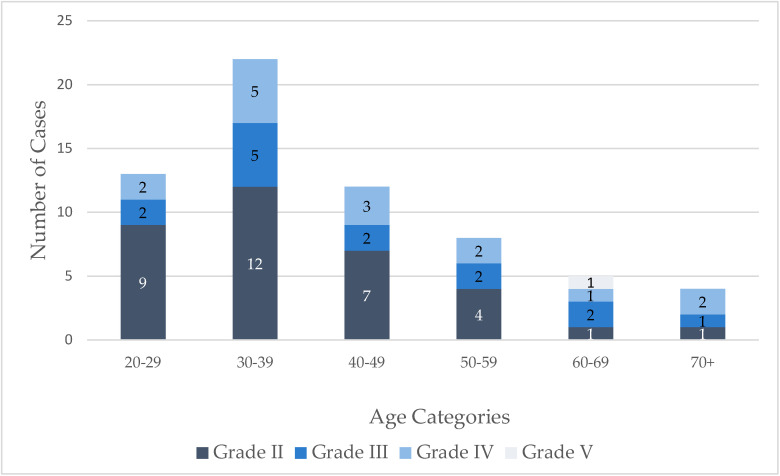
Burn patient group categorized by Dua grade and age.

In this study of 53 patients (64 eyes) with ocular chemical injuries, 23 were males (43.4%) and 30 were females (56.6%). The mean age was 41.84 ± 14.65 years. Most injuries were non-occupational accidents, primarily occurring within the household (81.1%) and during the spring (39.6%) and autumn (32.1%) seasons. The mean time to emergency department presentation was 5.74 hours. Alkali agents were the most common chemical cause (58.5%), and 90.6% of patients reported performing eye irrigation at home prior to arrival (Table 1). Low-grade injuries were predominant according to the Dua classification system (Figure 1).

In the burns group, the median horizontal cup-to-disk ratio (CDR) was 0.56 (range: 0.00–0.80), and the median vertical CDR was 0.55 (range: 0.00–0.78). No statistically significant difference was found (z = −0.821, *p* = 0.412).

Table 2 presents a comparison of OCT characteristics between healthy individuals and patients who sustained chemical eye burns. Overall RNFL thickness did not differ significantly between the groups (control group: 103.34 ± 7.81 µm (95% CI: 101.68–105.01 µm), burn patients: 102.20 ± 8.87 µm (95% CI: 99.99–104.42 µm); *p* = 0.646). However, nasal RNFL thickness was significantly reduced in the burn group (mean difference: −4.49 µm; *p* = 0.014), although this difference did not remain significant after Bonferroni correction (adjusted *p* = 0.056). Total GCL++ thickness did not show significant differences between the groups (control group: 105.03 ± 6.34 µm (95% CI: 103.68–106.38 µm), burn patients: 104.06 ± 8.14 µm (95% CI: 102.03–106.10 µm); *p* = 0.548). When comparing all measurements, a trend toward reduced RNFL and GCL++ thickness in burn patients was observed, although the differences were not statistically significant.

No statistically significant differences were observed in the total RNFL (t = −0.148, *p* = 0.883), total GCL++ (t = −1.423, *p* = 0.160), or other OCT-measured parameters based on the type of inflammatory agent (alkali or acid).

OCT asymmetry between the eyes was observed in 31.3% of subjects in the burn group. At the 3-month follow-up (i.e., six months after the injury), a reduction in the mean RNFL values was observed in the total, superior, nasal, and inferior quadrants; however, these changes were not statistically significant (all *p* > 0.05). Similarly, GCL++ measurements showed minimal variation between the two timepoints, with no statistically significant differences noted (Table 3).

Total RNFL thickness decreased by 1.89 µm (*p* = 0.349), with the greatest reduction in the inferior quadrant (4.38 µm). GCL++ thickness showed minimal change overall, with a mean decrease of 0.12 µm (*p* = 0.782) (Table 3).

Logistic regression analysis was employed to identify factors independently associated with RNFL and GCL++ thinning after ocular chemical injury. The Dua grade emerged as a significant predictor, with higher grades associated with increased odds of RNFL thinning (Table 4).

None of the evaluated factors showed a statistically significant association with GCL++ thinning, although age had the lowest *p*-value (0.085) (Table 5).

Compared to healthy controls, patients with severe chemical burns (Dua grade ≥ 3) exhibited reduced RNFL thickness across all quadrants. The most pronounced thinning was observed in the nasal (−6.35 µm, *p* = 0.007) and superior (−5.27 µm, *p* = 0.069) quadrants. After applying Bonferroni correction for multiple comparisons, only the difference in the nasal quadrant remained statistically significant (adjusted *p* = 0.035) (Table 6).

## 4. Discussion

In our study, the predominance of non-occupational, household-related accidents, particularly during spring and autumn, aligns with epidemiological data from similar studies reporting increased risk during periods of greater home activity and increased chemical use (e.g., cleaning agents) [19]. Household chemicals often contain alkaline agents, which were the most common cause of ocular burns in our study (58.5%). This is not surprising, as alkaline burns account for up to 68% of chemical burns in previous studies [19,20,21,22]. Interestingly, in contrast to most published literature reporting young male predominance in ocular chemical injuries, our cohort comprised a greater proportion of female patients (56.6%). The majority of incidents in our study were non-occupational and occurred at home, which is consistent with patterns of domestic exposure rather than workplace accidents, with the latter being more common in studies that report male predominance [19,20,21,22,23]. In our study, the mean age was 41.84 years, which is consistent with previous studies [19,24].

In this study, most subjects performed eye irrigation before arriving at the hospital. Although immediate irrigation after a burn is essential to interrupt ongoing exposure to the chemical agent and minimize damage to the anterior segment, it does not appear to reduce injury to the structures of the posterior pole. In animal experiments, post-burn eye irrigation did not affect the extent of damage to posterior segment structures. Furthermore, it did not influence pH changes in the anterior chamber, suggesting that pH regulation occurs via the uveoscleral pathway and is unaffected by irrigation [4]. Logistic regression analysis revealed that irrigation had no significant effect on RNFL or GCL++ thinning following ocular chemical injury in our study. No relationship was observed between the nature of the chemical and retinal thinning, although it is well known that alkali agents, due to their hydroxyl ions, induce saponification of fatty acids in cell membranes, allowing deeper penetration into the corneal stroma and causing damage to the cornea and anterior chamber structures. In contrast, acid injuries cause protein denaturation and tissue coagulation, which typically limit the depth of penetration [25]. Although alkali and acid burns differ in pathophysiology, our subgroup analysis revealed no statistically significant differences in retinal structural outcomes (RNFL, or GCL++ OCT parameters) between patients with alkali injuries and those with acid injuries.

The time from injury to arrival at the emergency department in our study ranged from 15 minutes to 48 hours. The authors conclude from animal studies that inflammatory processes causing irreversible and progressive neuroretinal tissue damage begin immediately after the burn. As early as six hours after the burn, the levels of inflammatory mediators are upregulated in the retina, and ganglion cell apoptosis is observed after twenty-four hours [4,8]. Moreover, administration of an antibody against TNF-α soon after the burn has been shown to provide significant protection to both the cornea and retina. In most experiments, preventive treatment with immunomodulators is applied fifteen minutes after the burn [26]. Our conclusion is that providing appropriate prophylactic treatment as soon as possible could reduce irreversible optic nerve axon and ganglion cell death, which is even more crucial than preserving the transparency of anterior segment structures, as the latter can be reconstructed later [7]. In our study, the most frequently used treatments were topical corticosteroids and antibiotic drops. C.H. Dohlman et al. claim that corticosteroids with other immunomodulators, if administered promptly, are markedly protective of the ganglion cells [3]. Given the mechanism of damage to GC and optic nerve axons, immunomodulatory therapy is the main focus. Prompt TNF-α blockade is considered a promising adjunct to existing therapeutic modalities [3,4]. A trend was observed in the burn group, with RNFL and GCL values being lower, although no statistically significant difference was found. According to data from the literature, GCL numbers and optic nerve axons in rabbit eyes declined significantly—to 40% and 30%, respectively—compared to measurements in the contralateral, non-burned eyes [8]. Asymmetry in OCT results between the burned and healthy eyes were found in about a third of the burn group subjects. Six months after the injury, thinning of the RNFL and GCL++ was observed in the burned eyes. Although this difference was not statistically significant, it exceeded the normal age-related variation described in the literature [27,28,29]. For example, Zhichao Wu et al. reported physiological RNFL thinning rates of approximately −0.54 μm/year in healthy adults [27]. Based on previous studies, the average annual thinning rate of the ganglion cell–inner plexiform layer (GCIPL) in healthy individuals is approximately −0.31 μm/year [28]. Another large-scale population-based study, the Singapore Chinese Eye Study, reported rates of −0.31 μm/year in males and −0.24 μm/year in females over a 6-year period [29]. In our study, total RNFL thickness decreased by 1.89 μm, and total GCL++ thickness showed a mean decrease of 0.12 μm over three months. In our study, the Dua grade emerged as a significant predictor, with higher grades associated with increased odds of RNFL thinning likely due to the more intense inflammation in the anterior segment.

To our knowledge, this is the first prospective in vivo study in humans to describe structural changes in the posterior segment of the eye following chemical corneal burns. Changes in the posterior pole following chemical injuries have previously been limited to clinical case reports [30]. This study has several limitations that should be considered when interpreting the results. First, the relatively small sample size may have reduced the statistical power to detect significant changes and associations. In addition, the short follow-up period may not fully reflect the progressive nature of retinal thinning after chemical ocular injury. A small degree of thinning of the nasal retinal nerve fiber layer (RNFL) (4.49 µm) was observed; however, this change may be within the range of physiological variability. Although this may indicate early or subclinical structural changes, its clinical significance remains unclear due to the lack of relevant functional data, such as visual field studies. The 6-month follow-up was limited to a subset of patients who had early interocular asymmetry at baseline. Such a targeted approach may introduce selection bias and potentially overestimate the degree of progression, thus limiting the generalizability of these findings. Larger, longitudinal studies are needed to confirm these results in a more representative population. This study did not include axial length measurements, limiting our ability to account for ocular magnification effects that may arise from corneal changes following chemical burns. In addition, the high prevalence of systemic corticosteroid use (98,1%) in this cohort may have influenced retinal thickness measurements. Although corticosteroids were used uniformly, their short-term effects on the retina are not well studied. The lack of an adequate untreated control group precludes the assessment of their independent effects, which may be a confounding factor. Finally, the timing of optical coherence tomography (OCT) imaging did not reflect the immediate post-traumatic period. Since the primary mechanism of injury is thought to occur within the first 24 hours, the lack of very early images may have limited the study’s ability to document the initial retinal response to chemical burns. Future studies incorporating larger, more diverse cohorts, extended follow-up periods, functional assessments, and early post-injury imaging are essential to better understand the onset, progression, and clinical implications of retinal changes in this patient population.

## 5. Conclusions

Gradually, more data is accumulating in relation to irreversible changes in the posterior pole of the eye, likely caused by inflammatory reactions stemming from chemical burns and other injuries to the anterior segment. However, assessing these changes in vivo has proven to be challenging due to the opacity of the anterior segment media. Furthermore, objective data regarding the condition of patients’ eyes prior to the injury is not always available. By elucidating the underlying causes of these observed changes, we can refine first aid protocols for eye injuries, thereby potentially preventing irreversible blindness. Consequently, further research in this area is crucial.

## Figures and Tables

**Table 1:** Demographics, clinical characteristics, and burn management. Abbreviations: ED, an emergency department.

	Number (n)	Percentage (%)
Demographics and Clinical Characteristics		
Sex		
Male	23	43.4
Female	30	56.6
Age (years)	41.84 ± 14.65	
Hospitalization		
Yes	10	18.9
No	43	81.1
Hospitalization days	5.10 ± 2.38	
Time from injury to ED arrival (hours)	5.74 (0.25–48)	
Occupation		
◦ Office clerk	11	20.8
◦ Farmer	2	3.8
◦ Worker	26	49.1
◦ Individual business	10	18.8
◦ Others	4	7.5
Season distribution		
Summer	8	15.1
Autumn	17	32.1
Winter	7	13.2
Spring	21	39.6
Place of injury		
Household	43	81.1
Workplace	10	18.9
Irrigation status before ED arrival		
Performed	48	90.6
Not performed	5	9.4
Part		
◦ Monocular injury	42	79.2
◦ Binocular injury	11	20.8
Chemical agent		
Alkali	31	58.5
◦ Acid	20	37.7
◦ Unknown	2	3.8
**Acute management of ocular burn**		
Oral doxycycline	10	18.9
Oral vitamin C	20	37.7
Topical corticosteroids	52	98.1
Therapeutic contact lens	5	9.4

**Table 2:** Comparison of OCT measurements between healthy individuals and patients after chemical burns. Values are presented as mean ± standard deviation (µm); values in parentheses indicate the range. Abbreviations: RNFL, retinal nerve fiber layer; GCL++, ganglion cell complex. * Bonferroni-corrected *p*-values are reported for multiple comparisons across the four RNFL quadrants (superior, nasal, inferior, temporal).

	Control Group (n = 87, eyes = 87)	Chemical Burn Group (n = 53, eyes = 64)	*p* Value	Corrected *p* Value *
**Patient Characteristics**				
Sex Male Female	41 (47.1%) 46 (52.9%)	23 (43.4%) 30 (56.6%)	*χ^2^ = 0.061, p = 0.805*	
Age (years)	45.49 ± 13.35 (21–87)	41.84 ± 14.65 (21–84)	*z = −1.867, p = 0.062*	
**OCT Characteristics**				
RNFL total (µm)	103.34 ± 7.81 (85–130)	102.20 ± 8.87 (78–123)	*t = −0.838, p = 0.404*	
RNFL superior (µm)	125.47 ± 12.41 (96–153)	124.94 ± 14.21 (78–159)	*t = −0.245, p = 0.806*	
RNFL nasal (µm)	82.08 ± 11.02 (59–104)	77.59 ± 10.97 (57–107)	*t = −2.477, p = 0.014*	0.056
RNFL inferior (µm)	134.59 ± 12.66 (104–173)	132.55 ± 14.34 (100–159)	*t = −0.925, p = 0.357*	
RNFL temporal (µm)	75.33 ± 12.37 (56–108)	72.33 ± 10.50 (52–97)	*t = −1.571, p = 0.118*	
GCL ++ total (µm)	105.03 ± 6.34 (91–126)	104.06 ± 8.14 (87–122)	*t = −0.825, p = 0.411*	
GCL ++ superior (µm)	104.01 ± 6.47 (91–123)	102.94 ± 8.14 (84–118)	*t = −0.903, p = 0.368*	
GCL ++ inferior (µm)	105.89 ± 6.63 (92–129)	105.12 ± 8.58 (89–125)	*t = −0.614, p = 0.540*	

**Table 3:** OCT characteristics of patients in the chemical burn group at 3-month and 6-month follow-up examinations. Values are presented as mean ± standard deviation (µm). Abbreviations: RNFL, retinal nerve fiber layer; GCL++, ganglion cell complex.

	3 Months	6 Months	*p* Value
**OCT Characteristics**			
RNFL total (µm)	98.11 ± 10.17	96.22 ± 8.33	*t = 1.681, p = 0.131*
RNFL superior (µm)	121.11 ± 13.89	119.89 ± 11.51	*t = 0.753, p = 0.473*
RNFL nasal (µm)	76.33 ± 13.14	73.44 ± 9.54	*t = 1.702, p = 0.127*
RNFL inferior (µm)	126.78 ± 15.18	122.4 ± 14.99	*t = 2.102, p = 0.069*
RNFL temporal (µm)	67.78 ± 8.91	68.67 ± 8.20	*t = −1.018, p = 0.338*
GCL ++ total (µm)	101.56 ± 5.56	101.44 ± 4.12	*t = 0.286, p = 0.782*
GCL ++ superior (µm)	100.33 ± 4.89	99.78 ± 4.06	*t = 0.743, p = 0.479*
GCL ++ inferior (µm)	102.78 ± 5.17	102.33 ± 4.21	*t = 0.645, p = 0.537*

**Table 4:** Logistic regression analysis of risk factors for RNFL thinning. Abbreviations: ED, an emergency department; RNFL, retinal nerve fiber layer.

	Odds Ratio	Confidence Interval (95%)	*p* Value
**Demographics and clinical characteristics**			
Sex			0.285
Age			0.595
Irrigation status before ED arrival (performed or not performed)			0.786
Insulting agent (acid or alkali)			0.813
Dua grade (I–VI)	4.816	1.103–21.030	0.037

**Table 5:** Logistic regression analysis of risk factors for GCL++ thinning. Abbreviations: ED, an emergency department; GCL++, ganglion cell complex.

	Odds Ratio	Confidence Interval (95%)	*p* Value
**Demographics and clinical characteristics**			
Sex			0.488
Age			0.085
Irrigation status before ED arrival (performed or not performed)			0.581
Insulting agent (acid or alkali)			0.186
Dua’s grade (I–VI)			0.280

**Table 6:** Comparison of OCT-derived RNFL thickness between healthy individuals and patients with severe chemical burns (Dua grade ≥ 3). Values are presented as mean ± standard deviation (µm); values in parentheses indicate the range. Abbreviations: RNFL, retinal nerve fiber layer. * Bonferroni-corrected *p*-values are reported for multiple comparisons across the RNFL quadrants.

	Control Group (n = 87)	Chemical Burn Group Dua grade ≥ 3 (n = 30)	*p* Value	Corrected *p* Value *
RNFL total (µm)	103.34 ± 7.81 (85–130)	100.17 ± 9.57 (78–121)	*t = −1.811, p = 0.073*	
RNFL superior (µm)	125.47 ± 12.41 (96–153)	120.20 ± 16.49 (78–159)	*t = −1.836, p = 0.069*	
RNFL nasal (µm)	82.08 ± 11.02 (59–104)	75.73 ± 10.88 (57–107)	*t = −2.729, p = 0.007*	0.035
RNFL inferior (µm)	134.59 ± 12.66 (104–173)	129.53 ± 15.54 (100–156)	*t = −1.775, p = 0.078*	
RNFL temporal (µm)	75.33 ± 12.37 (56–108)	72.20 ± 10.47 (52–95)	*t = −1.241, p = 0.217*	

## Data Availability

Data are contained within the article. The data that support the findings of this study are available from the corresponding author upon reasonable request. Further inquiries can be directed to the corresponding author.
